# Dental Society Work

**Published:** 1890-05

**Authors:** 


### Dental Society Work.

That dental association work has been one of the most important and efficient instrumentalities for elevating the dental profession none will deny. The greater the acquaintance and familiarity with the proper mode and manner of conducting dental societies the more beneficial will be the results. Perhaps the idea is generally entertained that it is quite sufficient for one to enter upon this variety of work after he has taken up and become fully established with the regular dental practice.

Experiment has already shown, however, that students of dentistry may, with great advantage, organize and conduct students' dental societies, and especially is this true of all students in dental colleges. The advantage to be derived from such associations is not to be lightly esteemed; they become familiar with the method of conducting society work, such as reading papers, discussing subjects, giving clinics, etc. It will also serve as a stimulus to more energetic study. The students in this way become better acquainted and learn the ability and strength of each other better than in any other way.

In several of the dental institutions in England such societies have been in operation for some considerable time, and the benefit accruing from them is universally conceeded. The papers read and discussed in these societies are from time to time published in the British journals, and they frequently compare favorably with papers presented in more pretentious bodies.

Students' dental societies have also been organized in some of the colleges in the United States. That connected with the New York College of Dentistry was one of the first, and is perhaps the most efficient students' society in this country. It has progressed so far as to issue and sustain a monthly journal—*The Student's Record*—which is very creditable indeed. Such societies have been formed in other colleges, but we know of none that has made quite the progress attained in this one.

There is no reason why the students of senior classes in all our colleges may not enter upon this work with decided advantage, and especially if they have had the right kind of training up to that point in their career. We hope that in the early future every dental college will have its students' association, and add that to the other facilities for their professional education.

